# Applicability of a duplex and four singleplex real-time PCR assays for the qualitative and quantitative determination of wild boar and domestic pig meat in processed food products

**DOI:** 10.1038/s41598-020-72655-7

**Published:** 2020-10-14

**Authors:** Maria Kaltenbrunner, Walter Mayer, Kirsten Kerkhoff, Rita Epp, Hermann Rüggeberg, Rupert Hochegger, Margit Cichna-Markl

**Affiliations:** 1 Department of Molecular Biology and Microbiology, Institute for Food Safety Vienna, Austrian Agency for Health and Food Safety, Spargelfeldstraße 191, 1220 Vienna, Austria; 2 Department of Analytical Chemistry, Faculty of Chemistry, University of Vienna, Währinger Straße 38, 1090 Vienna, Austria; 3 Impetus GmbH & Co. Bioscience KG, Fischkai 1, 27572 Bremerhaven, Germany

**Keywords:** PCR-based techniques, Genetic markers

## Abstract

Appropriate analytical methods are needed for the detection of food authentication. We investigated the applicability of a duplex real-time PCR assay targeting chromosome 1 and two singleplex real-time PCR assays targeting chromosome 9, both published recently, for the qualitative and quantitative determination of wild boar and domestic pig in processed food products. In addition, two singleplex real-time PCR assays targeting chromosome 7 were tested for their suitability to differentiate the two subspecies. Even by targeting the three genome loci, the probability of misclassification was not completely eliminated. Application of the real-time PCR assays to a total of 35 commercial meat products, including 22 goulash products, revealed that domestic pig DNA was frequently present, even in 14 out of 15 products declared to consist of 100% wild boar. Quantitative results obtained with the real-time PCR assays for wild boar (p < 0.001) and those for domestic pig (p < 0.001) were significantly different. However, the results obtained with the real-time PCR assays for wild boar (r = 0.673; p < 0.001) and those for domestic pig (r = 0.505; p = 0.002) were found to be significantly correlated. If the rules given in the paper are followed, the real-time PCR assays are applicable for routine analysis.

## Introduction

Food authentication is based on a variety of analytical methods, depending on the aspect of food adulteration one is interested in. Mislabeling of species in meat products is most commonly detected by DNA analysis^1, 2^. For the identification of pork, a number of methods based on polymerase chain reaction (PCR)—restriction fragment length polymorphism (RFLP) has been published^3–5^. Since PCR–RFLP is laborious and time consuming and not applicable to complex food products, it has mainly been replaced by singleplex^6–9^ and multiplex real-time PCR assays^10–14^. These assays usually allow the differentiation between pork and meat from other domesticated animals, e.g. beef, chicken or poultry, but are not applicable to distinguish pork from meat derived from wild boar. Differentiation between meat from domestic pig (*Sus scrofa domesticus*) and wild boar (*Sus scrofa scrofa*) is, however, necessary because in principle, both kinds of adulteration could occur: substitution of wild boar meat by pork and replacement of pork by wild boar meat. Since meat products derived from game animals are commonly more expensive than products from domesticated animals, some meat producers could be tempted to increase their profit by replacing (part of) wild boar meat by pork. However, at present some producers could also try to replace pork by wild boar meat due to an increase in pork prices, caused by slaughtering numerous pigs to reduce the chance of spreading of highly contagious African swine fever^15^.

Differentiation between pork and wild boar meat is a challenging task, because the genomes of wild boar and domestic pig are highly homologous^16, 17^. Thus, the number of subspecies-specific bases that can be targeted is very low. The common strategy is to make use of polymorphisms in genes that have been selected in the process of wild boar domestication. Genes associated with coat color, body composition, reproduction or behavior include *melanocortin 1 receptor* (*MC1R*), *v-kit Hardy-Zuckerman 4 feline sarcoma viral oncogene homolog* (*KIT*), *insulin like growth factor-2* (*IGF2*), *ryanodine receptor 1* (*RYR1*) and *nuclear receptor subfamily 6 group A member 1* (*NR6A1*)^18^.

Very recently, we developed a duplex and two singleplex real-time PCR assays for the differentiation between domestic pig and wild boar^19^. The assays are based on two nuclear DNA loci which had previously been reported to allow the differentiation between wild boar and domestic pig. The duplex real-time PCR assay, targeting the single nucleotide polymorphism (SNP) SNP g.299084751C>T in the *NR6A1* gene on chromosome 1, consists of assay_Chr1*W*_ for wild boar and assay_Chr1*D*_ for domestic pig. The two singleplex real-time PCR assays for wild boar and domestic pig, assay_Chr9*W*_ and assay_Chr9*D*_, target the SNP rs81416363 in an intergenic region on chromosome 9. Each of the assays was validated with regard to important analytical characteristics including selectivity, limit of detection (LOD), repeatability and robustness. By analyzing a total of 94 muscle tissue samples from 64 domestic pig and 30 wild boar individuals we found out that both the singleplex assays and the duplex assay led to some ambiguous results. However, we demonstrated that the assignment could be substantially improved by taking the results of both the duplex and the singleplex real-time PCR assays into account. With this approach, 91.5% of the 94 meat samples were classified correctly^19^.

Our previous study was restricted to unprocessed tissue samples from wild boar and domestic pig individuals. In the present study, we aimed at investigating the applicability of the real-time PCR assays for the qualitative and quantitative determination of wild boar and domestic pig meat in processed food products. Although the detection of undeclared meat species is sufficient to proof food adulteration, additional quantification allows the assessment if an undeclared species was added deliberately or is possibly contained due to contamination during food processing. A special case is Austria, where the proportion of wild boar meat and pork in commercial meat products is of interest because in “game meat” products, at least 38% of the meat content has to originate from game animals. In case the game species/subspecies is included in the product name, e.g. wild boar goulash, 38% of the meat content has to derive from the given species/subspecies^20^.

## Results and discussion

In order to investigate the applicability of the duplex and the singleplex real-time PCR assays for processed food products, we analyzed 34 commercial meat products declared to contain wild boar and one declared to contain domestic pig. The set of meat products consisted of 22 goulash samples, two roasts, one burger, 5 sausages, one pastry, three hams and one chips sample.

### Qualitative analysis

In 29 out of the 35 commercial meat products, wild boar was detected with both assays for wild boar, assay_Chr9*W*_ and assay_Chr1*W*_ (Table 1). In two products the wild boar content was < LOD (0.2%, w/w^19^) of assay_Chr9*W*_, in three products < LOD (2%, w/w^19^) of assay_Chr1*W*_. For the pork chips sample, both assays yielded a negative result. Although 15 out of the 22 goulash samples were declared to contain 100% wild boar, we detected domestic pig DNA in 13 samples with both assay_Chr9*D*_ (LOD: 2%, w/w^19^) and assay_Chr1*D*_ (LOD: 5%, w/w^19^). In total, domestic pig DNA was identified in 29 of the 35 commercial meat products with both assays for domestic pig. Wild boar ham 3 was the only sample in which domestic pig was neither detected with assay_Chr9*D*_ nor with assay_Chr1*D*_.

**Table 1:** Qualitative results obtained for 35 commercially available meat products (n = 4). Class: Classified, SD: Standard deviation, W: Wild boar, D: Domestic pig, ID: Identity

Sample	Declaration	Assay_Chr9*W*_			Assay_Chr9*D*_			Assay_Chr1*W*_			Assay_Chr1*D*_			Assay_Chr7_					Final result	
														Total pig		Domestic pig				
		Mean Ct	SD	Class W	Mean Ct	SD	Class D	Mean Ct	SD	Class W	Mean Ct	SD	Class D	Mean Ct	SD	Mean Ct	SD	ID	W	D
**Goulashes**																				
Wild boar goulash 1	100% wild boar	29.22	0.47	+	24.66	0.76	+	28.10	0.59	+	27.78	0.16	+	25.57	0.05	8.73	1.18	D	+	+
Wild boar goulash 2	100% wild boar	25.91	0.58	+	25.34	0.97	+	28.83	0.56	+	27.24	0.09	+	27	0.07	28.53	0.03	D	+	+
Wild boar goulash 3	100% wild boar	26.94	0.46	+	24.91	0.85	+	27.47	0.46	+	27.82	0.09	+	25.66	0.06	38.85	2.49	D	+	+
Wild boar goulash 4	100% wild boar	24.72	0.61	+	24.99	0.92	+	26.68	0.39	+	30.04	0.26	+	24.93	0.01	38.06	2.44	D	+	+
Wild boar goulash 5	100% wild boar	24.53	0.40	+	25.52	0.64	+	27.09	0.49	+	28.51	0.12	+	26.26	0.04	37.21	0.58	D	+	+
Wild boar goulash 6	100% wild boar	26.43	0.62	+	24.78	0.94	+	30.80	0.78	+	27.05	0.15	+	25.44	0.01	30.71	0.25	D	+	+
Wild boar goulash 7	100% wild boar	29.16	0.38	+	24.90	0.75	+	28.33	0.59	+	27.86	0.19	+	25.77	0.03	28.94	0.06	D	+	+
Wild boar goulash 8	100% wild boar	24.99	0.62	+	25.51	0.96	+	27.17	0.45	+	29.20	0.12	+	25.21	0.02	< LOD		no D	+	+
Wild boar goulash 9	100% wild boar	24.87	0.40	+	24.88	0.80	+	27.83	0.53	+	27.18	0.16	+	25.63	0.02	30.21	0.07	D	+	+
Wild boar goulash 10	100% wild boar	23.66	0.57	+	27.90	0.88	+	26.82	0.43	+	< LOD		−	25.95	0.01	< LOD		no D	+	−
Wild boar goulash 11	100% wild boar	24.96	0.55	+	25.42	0.84	+	27.64	0.46	+	28.03	0.16	+	25.81	0.02	< LOD		no D	+	+
Wild boar goulash 12	100% wild boar	25.14	0.51	+	25.28	0.90	+	28.02	0.75	+	28.47	0.20	+	25.79	0.06	< LOD		no D	+	+
Wild boar goulash 13	100% wild boar	26.75	0.73	+	25.09	1.04	+	31.11	0.74	+	27.04	0.22	+	25.42	0.02	< LOD		no D	+	+
Wild boar goulash 14	–	24.79	0.49	+	25.32	0.90	+	28.01	0.56	+	27.69	0.09	+	25.40	0	38.36	0.02	D	+	+
Wild boar goulash 15	–	29.06	0.85	+	24.65	1.16	+	27.55	0.40	+	28.21	0.14	+	25.79	0.04	< LOD		no D	+	+
Wild boar goulash 16	–	24.04	0.62	+	26.19	0.93	+	26.74	0.39	+	< LOD		−	25.50	0.04	< LOD		no D	+	−
Wild boar goulash 17	–	24.64	0.68	+	25.55	0.98	+	28.17	0.43	+	27.48	0.27	+	26.18	0.33	28.45	0.02	D	+	+
Wild boar goulash 18	100% wild boar	< LOD		−	24.51	1.14	+	26.42	0.13	+	< LOD		−	25.53	0	38.05	2.32	D	−	+
Wild boar goulash 19	100% wild boar	26.76	0.55	+	24.97	0.86	+	27.53	0.39	+	27.90	0.27	+	26.55	0.02	38.77		D	+	+
Wild boar goulash 20	–	24.22	0.56	+	26.62	0.79	+	28.13	0.48	+	27.62	0.20	+	24.38	0.04	38.79	0.18	D	+	+
Wild boar goulash 21	–	26.14	0.63	+	24.89	0.81	+	27.17	0.42	+	28.65	0.25	+	25.63	0.02	< LOD		no D	+	+
Wild boar goulash 22	–	25.62	0.96	+	25.30	1.01	+	27.72	0.36	+	28.15	0.21	+	26.07	0.05	35.34	0.09	D	+	+
**Burgers and roasts**																				
Wild boar roast 1	–	< LOD		−	24.51	0.91	+	27.47	0.49	+	27.62	0.10	+	25.38	0.01	< LOD		no D	+	+
Wild boar roast 2	Wild boar	24.60	0.68	+	25.65	0.85	+	28.18	0.29	+	27.70	0.27	+	26.71	0.05	< LOD		no D	+	+
Wild boar burger	–	26.03	0.55	+	24.76	1	+	27.66	0.52	+	27.15	0.09	+	26.37	0.09	31.02	0.11	D	+	+
**Sausages**																				
Game sausage 1	Deer, wild boar, roe deer (85%)	30.27	0.85	+	28.35	1.06	+	< LOD		−	31.41	0.45	+	32.44	0.1	31.15	0.23	D	+	+
Game sausage 2	Deer, wild boar, roe deer	24.59	0.66	+	27.78	0.99	+	27.57	0.26	+	30.24	0.26	+	29.51	0.22	31.68	0.07	D	+	+
Game salami	Wild boar	24.22	0.77	+	28.19	1.04	+	27.84	0.22	+	31.46	0.43	+	29.32	0.19	33.94	0.08	D	+	+
Wild boar sausage	Wild boar (70%), pig (21%)	29.86	0.81	+	24.51	1.01	+	< LOD		–	26.35	0.18	+	28.74	0.45	27.68	0.16	D	−	+
Game sausage 3	Deer, wild boar, roe deer (85%)	28.52	0.65	+	28.93	1.12	+	32.68	0.72	+	32.17	0.15	+	32.23	0.08	32.47	0.42	D	+	+
**Pastries**																				
Wild boar pastry	Wild boar (38%), pig, chicken	32.9	1.11	+	28.27	0.99	+	< LOD		–	31.46	0.20	+	33.86	0.08	38.69	0.70	D	−	+
Wild boar ham 1	–	23.51	0.7	+	32.78	1.24	+	26.86	0.27	+	< LOD		−	27.22	0.03	35.81	0.62	D	+	+
Wild boar ham 2	–	31.53	0.79	+	24.51	0.83	+	26.23	0.30	+	< LOD		−	25.05	0.02	33.72	0.46	D	+	+
Wild boar ham 3	–	21.41	0.13	+	< LOD		−	26.06	0.04	+	< LOD		−	27.38	0.04	< LOD		no D	+	−
Pork chips	Pig	< LOD		−	30.39	0.35	+	< LOD		−	29.94	0.16	+	32.25	0.31	35.78	0.33	D	−	+

Results for wild boar ham 2 were ambiguous. According to the results obtained with the assays targeting chromosome 9, the sample should originate from pig, whereas with the assays targeting chromosome 1 it was classified as wild boar. Most probably, the individual the meat is derived from does not show the genotype typical for wild boars, as demonstrated for several individuals by high resolution melting (HRM) analysis in our previous study^19^.

Since a high proportion of meat products was found to contain domestic pig although most of the products were declared to consist of 100% wild boar, we verified our results by analyzing the samples with two further singleplex real-time PCR assays, assay_Chr7*P*_ and assay_Chr7*D*_. Assay_Chr7*P*_ and assay_Chr7*D*_ are based on an insertion/deletion in a fragment of chromosome 7. Assay_Chr7*P*_ allows the detection of pig (wild boar and domestic pig), whereas assay_Chr7*D*_ is specific for domestic pig. Consequently, by taking the results of both assays into consideration, one is able to differentiate between wild boar and domestic pig. However, before the analysis of commercial meat products, we applied assay_Chr7*P*_ and assay_Chr7*D*_ to the analysis of DNA isolates from 64 domestic pig individuals, including 14 breeds and six cross-breeds, and a total of 30 wild boar samples from five different countries (Austria, Estonia, Germany, Romania and USA) (Suppl. Table S1). These DNA isolates had already been analyzed with assay_Chr9*W*_, assay_Chr9*D*_, assay_Chr1*W*_ and assay_Chr1*D*_ in our previous study^19^. As expected, with assay_Chr7*P*_, a positive result was obtained for each of the 64 domestic pig and 30 wild boar individuals. With assay_Chr7*D*_, almost all domestic pig breeds and cross-breeds led to an increase in the fluorescence signal. However, for three out of six Mangalica and all eight Turopolje individuals a negative result was obtained. The same Mangalica and Turopolje individuals had led to ambiguous results with the real-time PCR assays targeting chromosome 9 and chromosome 1, respectively**.**

For each of the 35 commercial meat products, a positive result was obtained with assay_Chr7*P*_, indicating that DNA from either wild boar, domestic pig or both subspecies was present (Table 1). By applying assay_Chr7*D*_, domestic pig DNA was detected in 24 out of the 35 products, including 10 goulash samples declared to contain 100% wild boar. This finding confirmed our results on the high frequency of domestic pig DNA in the set of commercial meat products obtained with assay_Chr9*D*_ and assay_Chr1*D*_. Furthermore, assay_Chr7*D*_ confirmed that wild boar ham 3 was the only sample which did not contain any domestic pig DNA.

By comparing the overall result obtained for each of the commercial meat products (Table 1) with the overall result obtained for each domestic pig and wild boar individual (Suppl. Table S1) we found out, that even by targeting the three different gene loci, the probability of misclassification was not completely eliminated. However, a meat sample containing one of the domestic pig breeds/crossbreeds investigated would hardly be classified as wild boar. The overall result obtained for wild boar goulash 10 and 16 (assay_Chr9*W,*_ assay_Chr9*D*_, assay_Chr1*W*_ and assay_Chr7*P*_ positive, assay_Chr1*D*_ and assay_Chr7*D*_ negative) was only identical with that obtained for one out of six Mangalica individuals (Mangalica 3). In addition, one wild boar individual from Austria (wild boar Austria 2) and two from Germany (Wiesbaden and Germany) led to the same overall result. The overall result obtained for wild boar goulash 11, 12, 13, 15 and 21 and wild boar roast 2 (assay_Chr9*W*_, assay_Chr9*D*_, assay_Chr1*W*_, assay_Chr1*D*_ and assay_Chr7*P*_ positive, assay_Chr7*D*_ negative) was only identical with that obtained for one wild boar individual (Germany, Perleberg). Wild boar roast 1 led to the same overall result as five out of eight Turopolje individuals. Game sausage 1, wild boar sausage and wild boar pastry showed the same overall result as Bentheim Black Pied pig 2 and three out of five Krškopolje breeds. The overall result obtained for wild boar ham 1 and 2 was only identical with that obtained for the wild boar individual USA 1. At present, more than 90% of all pigs slaughtered in Germany are crossbreeds from predominantly three pig breeds: German Edelschwein, German Landrace and Pietrain^21^. Thus, non-specific positive classifications of old pig breeds which are usually rare and marketed as delicacies are not limiting the applicability of the assays.

### Quantitative analysis

Next, we investigated the applicability of the duplex real-time PCR assay (assay_Chr1W_ and assay_Chr1D_) and the two singleplex real-time PCR assays (assay_Chr9W_ and assay_Chr9D_) for the determination of the content of wild boar and domestic pig in processed food products. (The assays targeting chromosome 7 are not suitable for obtaining quantitative information.) We pursued a relative quantification approach, based on relating the concentration of wild boar DNA (determined with assay_Chr9W_ or assay_Chr1W_) or domestic pig DNA (determined with assay_Chr9D_ or assay_Chr1D_) to the concentration of total meat DNA (determined with a reference real-time PCR assay published previously^22^). However, before we could apply the real-time PCR assays to the analysis of commercial meat products, we had to validate the assays regarding limit of quantification (LOQ) and recovery.

### Limit of quantification (LOQ)

We determined the LOQ by performing two series of experiments. In one series we used herring sperm DNA as background DNA. Herring sperm DNA is frequently used as non-target DNA in meat species authentication^19, 22, 23^. In the second series, we used domestic pig DNA as background DNA for assay_Chr9*W*_ and assay_Chr1*W*_ and wild boar DNA as background DNA for assay_Chr9*D*_ and assay_Chr1*D*_. The LOQ was defined as the lowest DNA concentration which could be determined with a relative standard deviation (RSD) ≤ 25%^24^. In one of our previous studies, LOQ values determined by using DNA mixtures were found to be similar to those determined by using DNA isolated from meat mixtures^25^. The advantage of using DNA mixtures is that mixtures with low proportions can be prepared without the necessity to weigh out high meat amounts. In addition, one can avoid inaccuracy due to the water content in low meat amounts.

**Figure 1:**
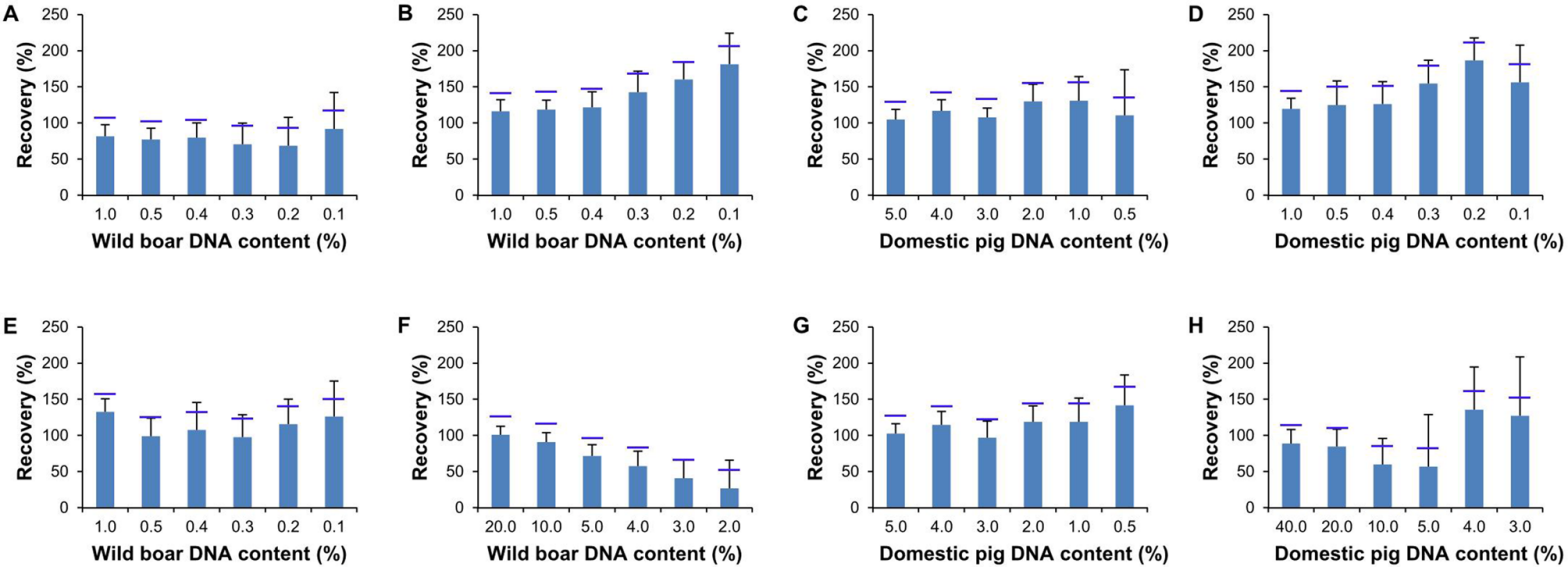
Determination of the LOQ by analyzing serial dilutions of wild boar or domestic pig DNA. (**A**)–(**D**) herring sperm DNA as background DNA, (**E**)–(**F**) domestic pig DNA as background DNA, (**G**)–(**H**) wild boar DNA as background DNA. (**A**),(**E**) assay_Chr9*W*_, (**B**),(**F**) assay_Chr1*W*_, (**C**),(**G**) assay_Chr9*D*_, and (**D**),(**H**) assay_Chr1*D*_. Column plots show mean recovery and relative standard deviation (RSD). Horizontal blue lines indicate a RSD of 25%.

When we determined the LOQ in herring sperm DNA as background DNA, the LOQ of assay_Chr9*W*_ (Fig. 1A) and assay_Chr1*W*_ (Fig. 1B) was found to be 0.4% (w/w) wild boar DNA, that of assay_Chr9*D*_ (Fig. 1C) and assay_Chr1*D*_ (Fig. 1D) 2% (w/w) and 1% (w/w) domestic pig DNA, respectively. In the presence of a surplus of domestic pig DNA, the LOQ of assay_Chr9*W*_ (Fig. 1E) and assay_Chr1*W*_ (Fig. 1F) was 0.5% (w/w) and 3.0% (w/w), respectively. However, since at 3.0% (w/w), recovery of assay_Chr1*W*_ was only 41%, we set the LOQ of assay_Chr1*W*_ to 5.0%. In the presence of an excess of wild boar DNA, the LOQ of assay_Chr9*D*_ (Fig. 1G) and assay_Chr1*D*_ (Fig. 1H) was found to be 2% (w/w) and 20.0% (w/w), respectively.

These data indicate that in the presence of DNA of the non-target subspecies, the LOQ of the assays targeting chromosome 1 was ten times higher than the LOQ of the assays targeting chromosome 9. The higher LOQ of assay_Chr1*W*_ and assay_Chr1*D*_ was most probably caused by signal suppression^19^. In our previous study, we demonstrated that at the same target DNA concentration, pure DNA extracts (either from wild boar or domestic pig) resulted in higher fluorescence signals (ΔRn values) than DNA mixtures containing both species (25:75 v/v)^19^.

### Recovery in meat extract mixtures

In order to determine the recovery of the duplex and singleplex real-time PCR assays, we analyzed DNA isolates from nine meat extract mixtures containing 0–70% (w/w) wild boar, 0–70% (w/w) domestic pig and 0–96% (w/w) cattle (Table 2). PCR was carried out in six replicates.

**Table 2:** Quantitative results obtained for meat extract mixtures and model sausages. PCR was carried out in 6 replicates.

Declared						Determined									
						Assay_Chr9_						Assay_Chr1_			
Wild boar	Domestic pig	Cattle	Red deer	Fallow deer	Roe deer	Wild boar		Domestic pig				Wild boar		Domestic pig	
Content (%)	Content (%)	Content (%)	Content (%)	Content (%)	Content (%)	Content (%) Mean ± SD	Recovery (%) Mean ± SD	Content (%) Mean ± SD	Recovery (%) Mean ± SD			Content (%) Mean ± SD	Recovery (%) Mean ± SD	Content (%) Mean ± SD	Recovery (%) Mean ± SD
**Meat extract mixtures**															
2	2	96	0	0	0	1 ± 0	72 ± 23	< LOQ				< LOD		< LOD	
5	5	90	0	0	0	3 ± 1	62 ± 16	4 ± 2	77 ± 45			< LOQ		< LOD	
10	10	80	0	0	0	8 ± 1	79 ± 5	9 ± 3	87 ± 34			9 ± 2	87 ± 18	< LOQ	
10	0	90	0	0	0	8 ± 1	82 ± 10	< LOD				9 ± 0	91 ± 4	< LOD	
0	10	90	0	0	0	< LOD		8 ± 2	84 ± 20			< LOD		< LOQ	
25	25	50	0	0	0	24 ± 3	97 ± 14	28 ± 9	113 ± 36			29 ± 1	116 ± 6	28 ± 3	114 ± 11
70	30	0	0	0	0	58 ± 4	83 ± 6	27 ± 10	90 ± 32			71 ± 10	101 ± 14	30 ± 3	101 ± 8
30	70	0	0	0	0	27 ± 4	91 ± 14	87 ± 17	124 ± 24			29 ± 4	97 ± 14	71 ± 10	102 ± 15
50	50	0	0	0	0	39 ± 1	79 ± 3	61 ± 22	123 ± 45			47 ± 5	94 ± 10	42 ± 5	84 ± 10
**Wild boar sausages**															
2	98	0	0	0	0	13 ± 1	671 ± 74	119 ± 23	122 ± 24			< LOD		95 ± 8	97 ± 8
10	90	0	0	0	0	26 ± 6	257 ± 56	98 ± 13	109 ± 15			< LOQ		109 ± 10	121 ± 11
25	75	0	0	0	0	32 ± 5	130 ± 22	73 ± 9	97 ± 13			24 ± 7	95 ± 29	88 ± 6	117 ± 8
38.5	61.5	0	0	0	0	49 ± 10	127 ± 25	14 ± 4	23 ± 6			39 ± 9	101 ± 23	74 ± 17	121 ± 28
50	50	0	0	0	0	38 ± 11	77 ± 22	38 ± 11	77 ± 22			31 ± 2	62 ± 4	46 ± 1	91 ± 3
**Game sausages**															
35	65	0	0	0	0	24 ± 2	69 ± 5	57 ± 12	88 ± 18			18 ± 3	51 ± 9	51 ± 9	78 ± 14
7	30	0	21	21	21	5 ± 0	73 ± 4	19 ± 6	63 ± 18			< LOQ		< LOQ	

Recoveries of assay_Chr9*W*_ were in the range from 62 to 97%. Low recoveries of 62% and 72% were obtained for meat extract mixtures containing 5% or 2% wild boar DNA, respectively. In case of assay_Chr9*D*_, a domestic pig content of 2% (w/w) was < LOQ. For the meat extract mixture containing 5% (w/w) domestic pig, a recovery of 60% was obtained, mixtures containing from 10 to 70% domestic pig resulted in recoveries between 84 and 124%. In meat extract mixtures containing 2% and 5% (w/w) wild boar, the wild boar DNA concentration was < LOQ of assay_Chr1*W*_. For meat extract mixtures containing 10–70% (w/w) wild boar, recoveries ranged from 87 to 116%. With assay_Chr1*D,*_ domestic pig DNA could not be quantified in a meat extract mixture containing 10% (w/w) domestic pig (< LOQ). For meat extract mixtures containing 25–70% (w/w) domestic pig, we obtained recoveries between 84 and 114%. A significant difference between recoveries was neither found for assay_Chr9*W*_ and assay_Chr1*W*_ (p = 0.356) nor for assay_Chr9*D*_ and assay_Chr1*D*_ (p = 0.653).

### Recovery in model game sausages

In addition to meat extract mixtures, we analyzed seven model sausages, including five wild boar and two game sausages. Six out of the seven sausages contained only meat from wild boar and domestic pig, whereas the seventh game sausage additionally contained meat from deer species (Table 2). The DNA isolates were analyzed in six PCR replicates.

In general, wild boar and domestic pig meat contents > 10% led to recoveries between 70 and 130%. The presence of low amounts of the target subspecies in the surplus of the non-target subspecies led to higher deviations from the theoretical value.

With assay_Chr9*W*_, we obtained recoveries between 69 and 130% for sausages with wild boar contents ranging from 25 to 50% (w/w). For the game sausage containing 7% (w/w) wild boar, 30% (w/w) domestic pig and 63% (w/w) deer meat, a recovery of 73% was obtained. For sausages consisting of 2% and 10% (w/w) wild boar and an excess of domestic pig (98% and 90% (w/w), respectively), the recoveries for wild boar were drastically too high (recovery 671% and 257%, respectively). High deviations from theoretical contents may be caused by differences in the composition, in particular the fat content, of meat tissues. We commonly try to overcome this problem by using calibration mixtures, carefully adjusted to composition and concentration of the samples^25–27^. The high recoveries obtained for wild boar in the presence of an excess of domestic pig is most probably caused by cross-reactivity of assay_Chr9*W.*_ For five sausages, assay_Chr9*D*_ led to recoveries between 77 and 122% for domestic pig contents between 30 and 98% (w/w). For the sausage containing 61.5% (w/w) domestic pig and 38.5% (w/w) wild boar, the domestic pig content was determined to be 14% (w/w) (recovery 23%). For the sausage containing 30% (w/w) domestic pig and 7% (w/w) wild boar, the recovery was 63%. Surprisingly, in both cases, the domestic pig content was underestimated. Due to the cross-reactivity with wild boar^19^, we actually expected the content to be overestimated. With assay_Chr1*W*_, recoveries between 51 and 101% were obtained for sausages with wild boar contents ranging from 25 and 50% (w/w). In the wild boar sausages containing 2% or 10% (w/w) wild boar in domestic pig, the concentration of wild boar DNA was < LOD and < LOQ, respectively. In addition, in the game sausage containing 7% (w/w) wild boar, 30% (w/w) domestic pig and 63% (w/w) deer, the wild boar concentration was < LOQ. With assay_Chr1*D*_, sausages with domestic pig contents ranging from 50 to 98% (w/w) resulted in recoveries between 78 and 121%. The domestic pig content in the sausage containing 7% (w/w) wild boar, 30% (w/w) domestic pig and 63% (w/w) deer was < LOQ. A significant difference between recoveries was neither found for assay_Chr9*W*_ and assay_Chr1*W*_ (p = 0.150) nor for assay_Chr9*D*_ and assay_Chr1*D*_ (p = 0.513).

### Analysis of commercially available meat products

**Figure 2:**
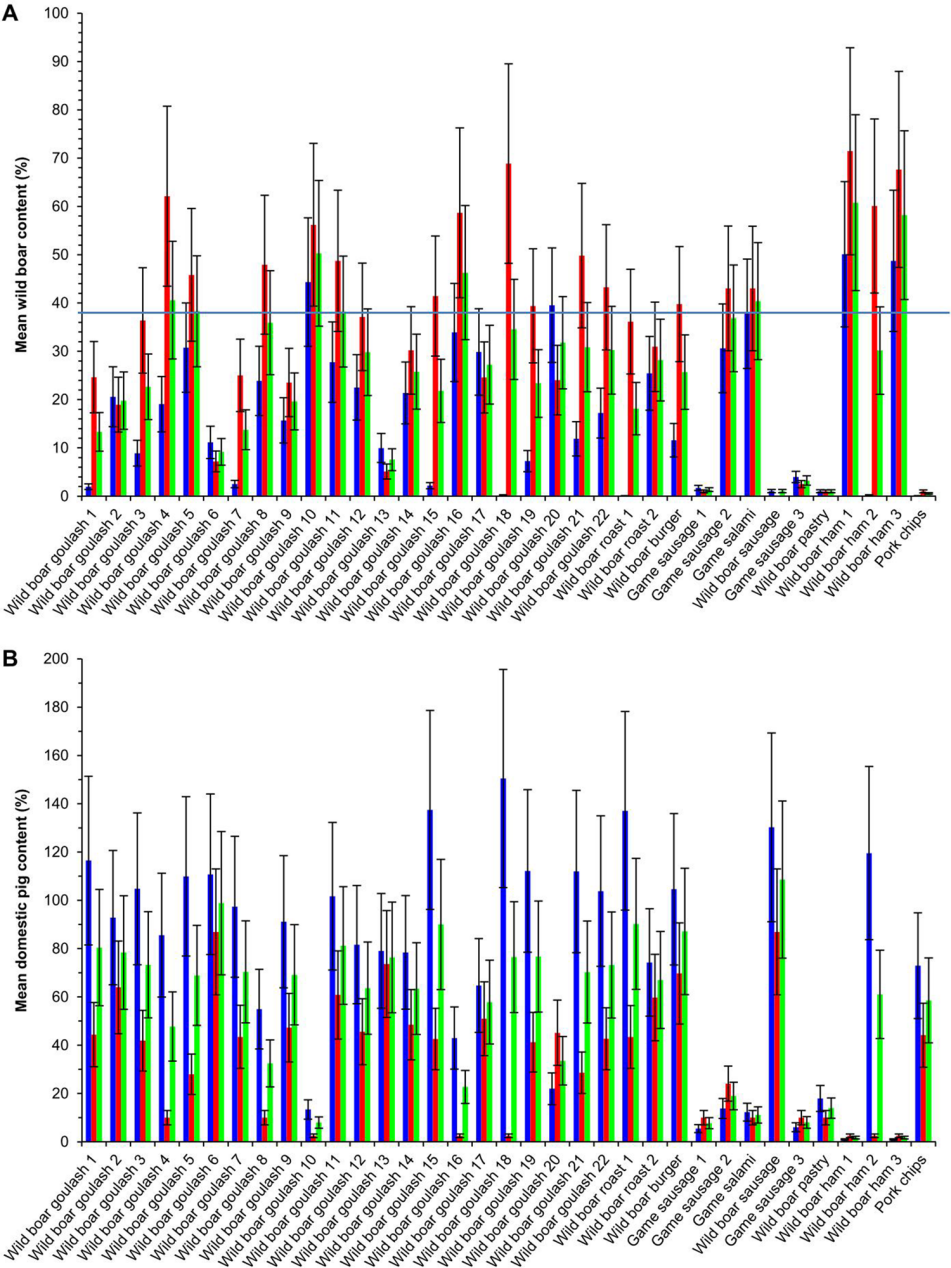
Quantitative results obtained for 35 commercial meat products. Mean content determined ± 30% error range (n ≥ 4). (**A**) wild boar (blue: assay_Chr9*W*_; red: assay_Chr1*W*_; green: overall mean) and (**B**) domestic pig (blue: assay_Chr9*D*_; red: assay_Chr1*D*_; green: overall mean). For results < LOD and < LOQ, LOD/2 and LOQ/2, respectively, are shown and were used for calculations. The horizontal blue line indicates a wild boar content of 38% (w/w).

Quantitative results obtained by analyzing the 35 commercial meat samples are summarized in Table 3. Figure 2A shows the mean values ± 30% error ranges for wild boar, determined with assay_Chr9*W*_ and assay_Chr1*W*_, Fig. 2B for domestic pig, determined with assay_Chr9*D*_ and assay_Chr1*D*_. Statistical analyses revealed significant differences between the results obtained for wild boar with assay_Chr9*W*_ and assay_Chr1*W*_ (p < 0.001) and also between the results for domestic pig obtained with assay_Chr9*D*_ and assay_Chr1*D*_ (p < 0.001). However, results obtained for wild boar with assay_Chr9*W*_ were significantly correlated with results obtained with assay_Chr1*W*_ (r = 0.673; p < 0.001). In addition, results obtained for domestic pig with assay_Chr9*D*_ significantly correlated with results obtained with assay_Chr1*D*_ (r = 0.505; p = 0.002).

**Table 3:** Quantitative results for 35 commercially available meat products (n ≥ 4).

Sample	Declaration	Assay_Chr9_		Assay_Chr1_		Final result	
		Wild boar content (%) Mean ± SD	Domestic pig content (%) Mean ± SD	Wild boar content (%) Mean ± SD	Domestic pig content (%) Mean ± SD	Wild boar content (%) ± SD	Domestic pig content (%) ± SD
**Goulash**							
Wild boar goulash 1	100% wild boar	2 ± 1	116 ± 24	25 ± 2	44 ± 4	14 ± 2	80 ± 24
Wild boar goulash 2	100% wild boar	21 ± 2	93 ± 16	19 ± 1	64 ± 4	20 ± 2	79 ± 16
Wild boar goulash 3	100% wild boar	9 ± 1	105 ± 22	36 ± 3	42 ± 5	23 ± 3	74 ± 23
Wild boar goulash 4	100% wild boar	19 ± 4	86 ± 6	62 ± 5	< LOQ	41 ± 6	86 ± 6
Wild boar goulash 5	100% wild boar	31 ± 6	110 ± 50	46 ± 7	28 ± 5	39 ± 9	69 ± 50
Wild boar goulash 6	100% wild boar	11 ± 2	111 ± 21	7 ± 1	87 ± 9	9 ± 2	99 ± 23
Wild boar goulash 7	100% wild boar	3 ± 1	97 ± 22	25 ± 3	43 ± 4	14 ± 3	70 ± 22
Wild boar goulash 8	100% wild boar	24 ± 5	55 ± 5	48 ± 4	< LOQ	36 ± 6	55 ± 5
Wild boar goulash 9	100% wild boar	16 ± 3	91 ± 13	24 ± 4	47 ± 7	20 ± 5	69 ± 15
Wild boar goulash 10	100% wild boar	44 ± 4	13 ± 4	56 ± 10	< LOD	50 ± 11	–
Wild boar goulash 11	100% wild boar	28 ± 4	102 ± 17	49 ± 10	61 ± 17	39 ± 11	82 ± 24
Wild boar goulash 12	100% wild boar	23 ± 3	82 ± 22	37 ± 5	46 ± 6	30 ± 6	64 ± 23
Wild boar goulash 13	100% wild boar	10 ± 2	79 ± 9	5 ± 1	74 ± 5	8 ± 2	77 ± 10
Wild boar goulash 14	–	21 ± 3	78 ± 18	30 ± 4	48 ± 6	26 ± 5	63 ± 19
Wild boar goulash 15	–	2 ± 1	137 ± 33	41 ± 17	43 ± 17	22 ± 17	90 ± 37
Wild boar goulash 16	–	34 ± 6	43 ± 11	59 ± 18	< LOD	47 ± 19	–
Wild boar goulash 17	–	30 ± 3	65 ± 6	25 ± 2	51 ± 5	28 ± 4	58 ± 8
Wild boar goulash 18	100% wild boar	< LOQ	150 ± 3	69 ± 7	< LOD	–	150 ± 3
Wild boar goulash 19	100% wild boar	7 ± 1	112 ± 22	39 ± 1	41 ± 5	23 ± 1	77 ± 23
Wild boar goulash 20	–	40 ± 10	22 ± 6	24 ± 2	45 ± 5	32 ± 10	34 ± 8
Wild boar goulash 21	–	12 ± 1	112 ± 16	50 ± 6	29 ± 1	31 ± 6	71 ± 16
Wild boar goulash 22	–	17 ± 0	104 ± 18	43 ± 9	43 ± 10	30 ± 9	74 ± 21
**Burgers and roasts**							
Wild boar roast 1	–	< LOD	137 ± 32	36 ± 4	43 ± 5	36 ± 4	90 ± 32
Wild boar roast 2	Wild boar	25 ± 4	74 ± 3	31 ± 6	60 ± 13	28 ± 7	67 ± 13
Wild boar burger	–	12 ± 1	105 ± 28	40 ± 8	70 ± 13	26 ± 8	88 ± 31
**Sausages**							
Game sausage 1	Deer, wild boar, roe deer (85%)	2 ± 1	6 ± 1	< LOD	< LOQ	2 ± 1	6 ± 1
Game sausage 2	Deer, wild boar, roe deer	31 ± 8	14 ± 5	43 ± 10	24 ± 9	37 ± 13	19 ± 10
Game salami	Wild boar	38 ± 3	12 ± 2	43 ± 9	< LOQ	41 ± 9	12 ± 2
Wild boar sausage	Wild boar (70%), pig (21%)	1 ± 0	130 ± 9	< LOD	87 ± 26	–	109 ± 28
Game sausage 3	Deer, wild boar, roe deer (85%)	4 ± 1	6 ± 2	< LOQ	< LOQ	3 ± 1	6 ± 2
**Pastries**							
Wild boar pastry	Wild boar (38%), pig, chicken	1 ± 0	18 ± 6	< LOD	< LOQ	–	18 ± 6
**Ham and chips**							
Wild boar ham 1	–	50 ± 6	< LOQ	71 ± 13	< LOD	61 ± 14	< LOQ
Wild boar ham 2	–	< LOQ	120 ± 30	60 ± 3	< LOD	60 ± 3	120 ± 30
Wild boar ham 3	–	49 ± 5	< LOQ	68 ± 6	< LOD	59 ± 8	–
Pork chips	Pig	< LOD	73 ± 9	< LOD	44 ± 5	–	59 ± 10

### Application in routine analysis

For this task, the following strategy should be pursued. To obtain qualitative information, each sample should be analysed with the six assays (assay_Chr9*W,*_ assay_Chr9*D*_, assay_Chr1*W,*_ assay_Chr1*D*_, assay_Chr7*P*_ and assay_Chr7*D*_)*.* If at least two of the three assays for the same subspecies lead to a positive result, the sample should be considered to contain the respective subspecies. If only one assay for this subspecies yields a positive result, the sample should be considered to not contain the subspecies. To determine the content of the detected subspecies, the application of the assay(s) is based on the qualitative results. If, e.g. domestic pig was detected with assay_Chr9*D*_ and assay_Chr7*D,*_ quantification should be done with assay_Chr9*D*_ (because the assays targeting chromosome 7 are not applicable for quantification). If, e.g. domestic pig was detected with both quantitative assays (assay_Chr9*D*_ and assay_Chr1*D*_), the mean value of the concentrations obtained with the assays should be used for authentication. If the concentration was determined to be < LOQ, it was substituted with half the LOQ.

By following these rules, the final qualitative (Table 1) and quantitative (Table 3) results were obtained. For three out of 35 samples (wild boar goulash 18, wild boar sausage and wild boar pastry) the wild boar content could not be determined. Only in one out of 15 goulash samples declared to contain only wild boar, domestic pig could not be detected. Thirteen goulash samples contained both wild boar and domestic pig, and in one goulash sample, only domestic pig was detected. According to the Codex Alimentarius Austriacus, in the goulash samples declared to contain wild boar, 38% (w/w) of the meat content should originate from wild boar. By taking into account a 30% error range, 13 out of the 22 goulash samples met the requirement of the Codex. In the wild boar pastry, wild boar could not be detected. We assume that the DNA was too degraded to be amplified. All burger, roast and ham samples met the requirement, whereas the wild boar sausage did not meet the requirement of the Codex.

## Conclusions

Both qualitative and quantitative determination of wild boar and domestic pig in processed food products turned out to be challenging. Even by targeting three different genome loci, the probability of misclassification was not completely eliminated. This is, however, not mainly caused by cross-reactivity of the real-time PCR assays. The major problem is that special pig breeds and cross-breeds show one or even two alleles commonly found in wild boar, whereas certain wild boar individuals are heterozygous or even homozygous for the allele usually occurring in domestic pig^19^. This obviously may also lead to discrepancies in quantitative results obtained by real-time PCR assays targeting different genome loci. Another well-known difficulty in the quantification of meat species in food by real-time PCR is the calculation of the meat content (w/w) from the target DNA concentration determined. Our strategy to overcome this problem is to use calibration mixtures similar to the analyzed sample in both the composition and concentration of the meat species of interest. In previous studies, this strategy was very successful^25–27^.

## Materials and methods

### Samples

Meat from wild boar individuals was collected in Austria and Germany, meat from domestic pig and cattle was purchased from local suppliers. All samples originated from lean muscle meat.

Meat extract mixtures were prepared by mixing the extracts from meat flesh of the respective animal species prior to DNA isolation. Model sausages were produced according to the guidelines of the Codex Alimentarius Austriacus^20^. “Wild boar sausages” containing different amounts of wild boar [2%, 10%, 25%, 38.5% or 50% (w/w)] and domestic pig [[98%, 90%, 75%, 61.5% or 50% (w/w), respectively] were produced at the Austrian Agency for Health and Food Safety (AGES, Vienna, Austria). Two other game sausages were produced at the Higher Technical College for Food Technology Hollabrunn (Hollabrunn, Austria). One game sausage contained 65% (w/w) domestic pig (30% bacon and 35% meat) and 35% (w/w) wild boar. The other game sausage consisted of 21% red deer, 21% fallow deer, 21% roe deer, 7% wild boar and 30% (w/w) bacon (domestic pig). Both sausages also contained typical sausage ingredients like nitrite curing salt (28 g/kg sausage), dextrose (3 g/kg sausage) and sucrose (2 g/kg sausage). In addition, the first one contained 54 g and the latter one 27 g of an allergen mix (celery, white/brown/black mustard, sesame, soy, wheat, egg powder and milk powder) per 15 kg.

34 food products declared to contain wild boar meat were purchased from supermarkets in Austria and Germany, comprising goulash (n = 22), roast (n = 2), burger (n = 1), sausage (n = 5), pastry (n = 1) and ham (n = 3). Furthermore, chips (n = 1) declared to contain domestic pig were analyzed.

### DNA isolation

Sausages were homogenized in a knife mill (Retsch GM 200, Retsch, Haan, Germany). Muscle meat samples were cut into small pieces and used as-is. Commercial meat products were homogenized or cut into small pieces. 1–2 g of the sample material (meat, model sausage or commercial game product) were weighed out and 10 mL CTAB extraction solution (2% (w/v) CTAB, 1.4 M NaCl, 0.1 M Tris, 0.02 M EDTA, adjusted to pH 8.0 with 4 M HCl, autoclaved) and 80 µL proteinase K solution (1 mg/mL) were added. Lysis was carried out in an incubator (Unihood 750, Uniequip, Martinsried, Germany) at 50 °C under shaking (Intelli-Mixer RM-2L, LTF Labortechnik, Wasserburg, Germany) until the starting material was dissolved (at least 4 h). DNA was isolated at least twice from each lysis batch according to a previously published CTAB protocol^28^. To determine the DNA concentration and purity of the obtained DNA isolates, the absorbance was measured at 260 nm and 280 nm using a QIAxpert spectrophotometer (Qiagen, Hilden, Germany). The DNA isolates were stored at − 20 °C.

### Real-time PCR

The two singleplex assays, assay_Chr9*W*_ and assay_Chr9*D*_, and the duplex real-time PCR assay, consisting of assay_Chr1*W*_ and assay_Chr1*D*_, were developed and validated previously^19^. Primer and probe sequences, target genes, accession numbers (NCBI GenBank accession number), final concentrations of primers and probes used in the PCR assays and amplicon lengths are given in Suppl. Table S2. Primers and probes were synthesized by Sigma Aldrich and Eurogentec (Seraing, Belgium), respectively.

Real-time PCR was performed in an optical 96-well reaction plate (0.2 mL, Applied Biosystems, Foster City, CA, USA) sealed with optical adhesive film (Applied Biosystems) on the ABI 7500 Real-time PCR System (Applied Biosystems). The reaction mix (total volume 25 µL) consisted of 12.5 µL of QuantiTect Multiplex PCR NoROX Master Mix (Qiagen), 2.5 µL of ultrapure water, 5 µL of 5 × primer/probe mix and 5 µL DNA isolate (DNA concentration between 5 and 20 ng/µL). For using the QuantiTect Multiplex PCR NoROX Master Mix on the ABI 7500 Real-time PCR System, 1.8 mL of the mix was pre-mixed with 2 µL ROX Reference Dye (25 µM) (Invitrogen by Life Technologies, Carlsbad, CA, USA). The final ROX concentration per PCR reaction was 14 nM. The temperature program was initiated with a denaturation step at 95 °C for 15 min, followed by 40 cycles at 94 °C for 1 min and 60 °C for 1 min.

DNA isolates from the 35 commercial food products were diluted with water to a concentration of 10 ng/µL. In addition to the samples, positive controls were added to each PCR plate. The positive control for assay_Chr9*W*_ and assay_Chr9*D*_ contained 0.2% (w/w) wild boar DNA, 0.5% (w/w) domestic pig DNA and 99.3% (w/w) herring sperm DNA. In case of assay_Chr1*W*_ and assay_Chr1*D*_, a mixture consisting of 2% (w/w) wild boar DNA, 5% (w/w) domestic pig DNA and 93% (w/w) herring sperm DNA was used.

### Limit of quantification (LOQ)

The LOQ was determined as recommended by the European Network of GMO Laboratories^24^. The LOQ was defined as the lowest concentration which could be determined with a RSD ≤ 25%. Assay_Chr9*W*_ and assay_Chr1*W*_ were calibrated with a mixture (20 ng/µL) of 25% (w/w) wild boar DNA and 75% (w/w) herring sperm DNA (non-target DNA), assay_Chr9*D*_ and assay_Chr1*D*_ with a mixture (20 ng/µL) of 25% (w/w) domestic pig DNA and 75% (w/w) herring sperm DNA (non-target DNA). The mixtures were analyzed both undiluted and serially diluted (1:4; 1:16; 1:64; 1:256; 1:1,024; in water) in triplicates. The following DNA mixtures (5 ng/µL) were used: assay_Chr9*W*_ and assay_Chr1*W*_: 1%, 0.5%, 0.4%, 0.3%, 0.2% and 0.1% (w/w) wild boar DNA in herring sperm DNA; assay_Chr9*D*_: 5%, 4%, 3%, 2%, 1% and 0.5% (w/w) domestic pig DNA in herring sperm DNA and assay_Chr1*D*_: 1%, 0.5%, 0.4%, 0.3%, 0.2% and 0.1% (w/w) domestic pig DNA in herring sperm DNA.

In addition, the LOQ of the assays for wild boar and domestic pig was determined by using domestic pig and wild boar DNA as background, respectively.

Assay_Chr9*W*_ and assay_Chr1*W*_ were calibrated with a mixture (20 ng/µL) containing 25% (w/w) wild boar DNA and 75% (w/w) domestic pig DNA, assay_Chr9*D*_ with a mixture (20 ng/µL) containing 25% (w/w) domestic pig DNA and 75% (w/w) wild boar DNA and assay_Chr1*D*_ with a mixture (20 ng/µL) containing 50% (w/w) domestic pig DNA and 50% (w/w) wild boar DNA. The mixtures were analyzed undiluted and serially diluted (1:4; 1:16; 1:64; 1:256; 1:1,024; in water) in triplicates. The following DNA mixtures (5 ng/µL) were used: assay_Chr9*W*_: 1%, 0.5%, 0.4%, 0.3%, 0.2% and 0.1% (w/w) wild boar DNA in domestic pig DNA; assay_Chr9*D*_: 5%, 4%, 3%, 2%, 1% and 0.5% (w/w) domestic pig DNA in wild boar DNA; assay_Chr1*W*_: 20%, 10%, 5%, 4%, 3% and 2% (w/w) wild boar DNA in domestic pig DNA; assay_Chr1*D*_: 40%, 20%, 10%, 5%, 4% and 3% (w/w) domestic pig DNA in wild boar DNA.

### Quantitative analysis of samples

The content of wild boar and domestic pig in meat extract mixtures, model game sausages and commercial meat products was determined relatively. The DNA isolates were analyzed with the respective sub-species specific real-time PCR assay (assay_Chr9*W*_, assay_Chr9*D*_, assay_Chr1*W*_ or assay_Chr1*D*_) and a reference real-time PCR assay published previously^22^. The reference real-time PCR assay allowed the determination of the total DNA concentration of mammalian and poultry species in the isolate.

For obtaining quantitative results, the real-time PCR assays had to be calibrated. For the analysis of meat extract mixtures, a meat extract mixture containing 25% (w/w) wild boar, 50% (w/w) domestic pig and 25% (w/w) cattle was used for calibration. PCR was carried out in six replicates. For the quantitative analysis of commercial meat products, the assays were calibrated with a meat extract mixture consisting of 30% (w/w) wild boar and 70% (w/w) domestic pig. PCR was carried out in four replicates.

The calibration mixtures were adjusted to a DNA concentration of 20 ng/µL and serially diluted in water (1:4; 1:16; 1:64; 1:256 and 1:1,024). DNA isolates from samples were diluted to a DNA concentration of 5 ng/µL. The content of wild boar and domestic pig was assessed by using the following equations:

1 $${c}_{DNA~wild~boar/domestic~pig}\left(\frac{\mathrm{ng}}{\mathrm{\mu L}}\right)={10}^{\frac{{Ct}_{wild~boar/domestic~pig}-{d}_{wild~boar/domestic~pig}}{{slope}_{wild~boar/domestic~pig}}}$$

2 $${c}_{DNA~total~meat}\left(\frac{\mathrm{ng}}{\mathrm{\mu L}}\right)={10}^{\frac{{Ct}_{ref.}-{d}_{ref.}}{{slope}_{ref.}}}$$

3 $${content}_{wild~boar/domestic~pig} \left(\mathrm{\%}\right)=\frac{{c}_{DNA~wild~boar/domestic~pig}\left(\frac{\mathrm{ng}}{\mathrm{\mu L}}\right)}{{c}_{DNA~total~meat}\left(\frac{\mathrm{ng}}{\mathrm{\mu L}}\right)}\cdot 100$$

where c_DNA_ _wild_ _boar/domestic_ _pig_, c_DNA_ _total_ _meat_ are the concentrations of wild boar DNA, domestic pig DNA and total meat DNA, respectively; Ct_wild_ _boar/domestic_ _pig_, Ct_ref._ are the Ct values obtained with the wild boar-/domestic pig-specific assay and the reference real-time PCR assay, respectively; d_wild_ _boar/domestic_ _pig_, d_ref._ are the intercepts of the standard curves of the wild boar-/domestic pig-specific assay and the reference real-time PCR assay, respectively; and slope_wild_ _boar/domestic_ _pig_, slope_ref._ are the slopes of the calibration curves of the wild boar-/domestic pig-specific assay and the reference real-time PCR assay, respectively.

In case of meat extract mixtures and model sausages, the recovery was calculated by referring the wild boar/domestic pig content determined by real-time PCR to the theoretical wild boar/domestic pig content using the following equation:

4 $$Recovery~R (\%)=\frac{determined~wild~boar/domestic~pig~content (\%)\cdot 100}{theoretical~wild~boar/domestic~pig~content (\%)}$$

### Qualitative analysis carried out with a real-time PCR targeting a fragment of chromosome 7

Experimental details are given in the Supplementary Material.

### Statistical analysis

Statistical analyses were carried out with IBM SPSS Statistics Version 26.0. Concentrations < LOD and < LOQ were substituted with default values, namely half the LOD and half the LOQ, respectively, as proposed previously^29^. The T test was used to test for significant differences between recoveries of PCR assays. Pearson´s correlation coefficient was used to assess the relationship between results obtained with different PCR assays. In all cases, a *p* value < 0.05 (two-sided) was considered significant.

### Use of experimental animals

We did not perform experiments on live vertebrates, we only analysed muscle meat which we obtained from dead/slaughtered animals.

## Supplementary information

Supplementary file1
