# Supplementary material for: Applicability of a duplex and four singleplex real-time PCR assays for the qualitative and quantitative determination of wild boar and domestic pig meat in processed food products
Source: Sci Rep. 2020 Oct 14;10:17243. doi: 10.1038/s41598-020-72655-7 (PMC7560752; doi:10.1038/s41598-020-72655-7)
Supplement: Supplementary file 1 — Supplementary file1 [file 41598_2020_72655_MOESM1_ESM.docx]

**Applicability of a duplex and four singleplex real-time PCR assays for the qualitative and quantitative determination of wild boar and domestic pig meat in processed food products**

Maria Kaltenbrunner^1,2^, Walter Mayer^1^, Kirsten Kerkhoff^3^, Rita Epp^3^, Hermann Rüggeberg^3^, Rupert Hochegger^1,*^ and Margit Cichna-Markl^2,*^

^1^Austrian Agency for Health and Food Safety, Institute for Food Safety Vienna, Department of Molecular Biology and Microbiology, Spargelfeldstraße 191, 1220 Vienna, Austria

^2^Department of Analytical Chemistry, Faculty of Chemistry, University of Vienna, Währinger Straße 38, 1090 Vienna, Austria

^3^Impetus GmbH & Co. Bioscience KG, Fischkai 1, 27572 Bremerhaven, Germany

***Corresponding authors**

Rupert Hochegger: [rupert.hochegger@ages.at](mailto:rupert.hochegger@ages.at)

Margit Cichna-Markl: [margit.cichna@univie.ac.at](mailto:margit.cichna@univie.ac.at)

**The co-authors e-mail addresses:**

Maria Kaltenbrunner: [maria.kaltenbrunner@univie.ac.at](mailto:maria.kaltenbrunner@univie.ac.at)

Walter Mayer: [walter.mayer@ages.at](mailto:walter.mayer@ages.at)

Kirsten Kerkhoff: [k.kerkhoff@impetus-bioscience.de](mailto:k.kerkhoff@impetus-bioscience.de)

Rita Epp: [r.epp@impetus-bioscience.de](mailto:r.epp@impetus-bioscience.de)

Hermann Rüggeberg: [h.rueggeberg@impetus-bioscience.de](mailto:h.rueggeberg@impetus-bioscience.de)

**Supplementary Table 1** Comparison of the qualitative results obtained for **A)** 64 domestic pig individuals and **B)** 30 wild boar individuals with assay_Chr9_*_W_*, assay_Chr9_*_D_*, assay_Chr1_*_W_*, assay_Chr1_*_D,_* assay_Chr7_*_P_* and assay_Chr7_*_D_* (n = 4). Results obtained with assay_Chr9_*_W_*, assay_Chr9_*_D_*, assay_Chr1_*_W_* and assay_Chr1_*_D_* were published previously*^19^*. Correct classifications are highlighted in green, incorrect classifications in red.

**A)**

|  |  | **Assay_Chr9_*_W_*** |  | **Assay_Chr9_*_D_*** |  | **Assay_Chr1_*_W_*** |  | **Assay_Chr1_*_D_*** |  | **Assay_Chr7_*_P_*** |  | **Assay_Chr7_*_D_*** |
| --- | --- | --- | --- | --- | --- | --- | --- | --- | --- | --- | --- | --- |
| **Species** |  | **Class W** |  | **Class D** |  | **Class W** |  | **Class D** |  | **Class P** |  | **Class D** |
| Angeln Saddleback 1 |  | - |  | + |  | - |  | + |  | + |  | + |
| Angeln Saddleback 2 |  | - |  | + |  | - |  | + |  | + |  | + |
| Bentheim Black Pied pig 1 |  | - |  | + |  | - |  | + |  | + |  | + |
| Bentheim Black Pied pig 2 |  | + |  | + |  | - |  | + |  | + |  | + |
| Cinta Senese 1^a^ |  | - |  | + |  | - |  | + |  | + |  | + |
| Cinta Senese 2^a^ |  | - |  | + |  | - |  | + |  | + |  | + |
| Cinta Senese 3^a^ |  | - |  | + |  | - |  | + |  | + |  | + |
| Cinta Senese 4^a^ |  | - |  | + |  | - |  | + |  | + |  | + |
| Cinta Senese 5^a^ |  | - |  | + |  | - |  | + |  | + |  | + |
| Cinta Senese 6^a^ |  | - |  | + |  | - |  | + |  | + |  | + |
| Cinta Senese 7^a^ |  | - |  | + |  | - |  | + |  | + |  | + |
| Cinta Senese 8^a^ |  | - |  | + |  | - |  | + |  | + |  | + |
| Cinta Senese 9^a^ |  | - |  | + |  | - |  | + |  | + |  | + |
| Cinta Senese 10^a^ |  | - |  | + |  | - |  | + |  | + |  | + |
| Cinta Senese 11^a^ |  | - |  | + |  | - |  | + |  | + |  | + |
| Cinta Senese 12^a^ |  | - |  | + |  | - |  | + |  | + |  | + |
| Duroc 1 |  | - |  | + |  | - |  | + |  | + |  | + |
| Duroc 2 |  | - |  | + |  | - |  | + |  | + |  | + |
| Duroc 3 |  | - |  | + |  | - |  | + |  | + |  | + |
| Duroc 4 |  | - |  | + |  | - |  | + |  | + |  | + |
| Duroc 5 |  | - |  | + |  | - |  | + |  | + |  | + |
| Duroc 6 |  | - |  | + |  | - |  | + |  | + |  | + |
| Duroc 7 |  | - |  | + |  | - |  | + |  | + |  | + |
| G Edelschwein 1^b^ |  | - |  | + |  | - |  | + |  | + |  | + |
| G Edelschwein 2 |  | - |  | + |  | - |  | + |  | + |  | + |
| G Edelschwein 3 |  | - |  | + |  | - |  | + |  | + |  | - |
| Iberian pig |  | - |  | + |  | - |  | + |  | + |  | + |
| Krškopolje 1 |  | - |  | + |  | - |  | + |  | + |  | + |
| Krškopolje 2 |  | - |  | + |  | - |  | + |  | + |  | + |
| Krškopolje 3 |  | + |  | + |  | - |  | + |  | + |  | + |
| Krškopolje 4 |  | + |  | + |  | - |  | + |  | + |  | + |
| Krškopolje 5 |  | + |  | + |  | - |  | + |  | + |  | + |
| G Landrace 1 |  | - |  | + |  | - |  | + |  | + |  | + |
| G Landrace 2 |  | - |  | + |  | - |  | + |  | + |  | + |
| G Landrace 3 |  | - |  | + |  | - |  | + |  | + |  | + |
| Mangalica 1 |  | + |  | - |  | - |  | + |  | + |  | - |
| Mangalica 2 |  | + |  | + |  | - |  | + |  | + |  | - |
| Mangalica 3 |  | + |  | + |  | + |  | - |  | + |  | - |
| Mangalica 4 |  | - |  | + |  | - |  | + |  | + |  | + |
| Mangalica 5 |  | - |  | + |  | - |  | + |  | + |  | + |
| Mangalica 6 |  | - |  | + |  | - |  | + |  | + |  | + |
| Pietrain |  | - |  | + |  | - |  | + |  | + |  | + |
| Husum Red Pied 1 |  | - |  | + |  | - |  | + |  | + |  | + |
| Husum Red Pied 2^b^ |  | - |  | + |  | - |  | - |  | + |  | + |
| Saddleback 1 |  | - |  | + |  | - |  | + |  | + |  | + |
| Saddleback 2 |  | - |  | + |  | - |  | + |  | + |  | + |
| Swabian-Hall swine |  | - |  | + |  | - |  | + |  | + |  | + |
| Turopolje 1 |  | - |  | + |  | + |  | + |  | + |  | - |
| Turopolje 2 |  | - |  | + |  | + |  | + |  | + |  | - |
| Turopolje 3 |  | - |  | + |  | + |  | + |  | + |  | - |
| Turopolje 4 |  | - |  | + |  | - |  | + |  | + |  | - |
| Turopolje 5 |  | - |  | + |  | + |  | + |  | + |  | - |
| Turopolje 6 |  | - |  | + |  | + |  | + |  | + |  | - |
| Turopolje 7 |  | - |  | + |  | - |  | + |  | + |  | - |
| Turopolje 8 |  | - |  | + |  | + |  | - |  | + |  | - |
| G Edelschwein x G Landrace |  | - |  | + |  | - |  | + |  | + |  | + |
| G Landrace x G Edelschwein |  | - |  | + |  | - |  | + |  | + |  | + |
| Duroc x Landrace |  | - |  | + |  | - |  | + |  | + |  | + |
| Pietrain x (G Landrace + G Edelschwein) |  | - |  | + |  | - |  | + |  | + |  | + |
| Duroc*Hampshire 1^b^ |  | - |  | + |  | - |  | + |  | + |  | + |
| Duroc*Hampshire 2^b^ |  | - |  | + |  | - |  | + |  | + |  | + |
| Duroc*Pietrain 1 |  | - |  | + |  | - |  | + |  | + |  | + |
| Duorc*Pietrain 2 |  | - |  | + |  | - |  | + |  | + |  | + |
| Pig (Supermarket) |  | - |  | + |  | - |  | + |  | + |  | + |
| Class.… classification of the sample; W… wild boar; D… domestic pig; P… sum of wild boar and domestic pig; G… German; ^a^… meat taken from the loin; ^b^…sample taken from punching the ear tag | | | | | | | | | | | | |

**B)**

|  |  | **Assay_Chr9_*_W_*** |  | **Assay_Chr9_*_D_*** |  | **Assay_Chr1_*_W_*** |  | **Assay_Chr1_*_D_*** |  | **Assay_Chr7_*_P_*** |  | **Assay_Chr7_*_D_*** |
| --- | --- | --- | --- | --- | --- | --- | --- | --- | --- | --- | --- | --- |
| **Species** |  | **Class W** |  | **Class D** |  | **Class W** |  | **Class D** |  | **Class P** |  | **Class D** |
| Wild boar (Upper Austria 1) |  | + |  | - |  | + |  | - |  | + |  | - |
| Wild boar (Upper Austria 2) |  | + |  | - |  | + |  | - |  | + |  | - |
| Wild boar (Lower Austria 1) |  | + |  | - |  | + |  | + |  | + |  | - |
| Wild boar (Lower Austria 2) |  | + |  | - |  | + |  | - |  | + |  | - |
| Wild boar (Lower Austria 3) |  | + |  | - |  | + |  | - |  | + |  | - |
| Wild boar (Lower Austria 4) |  | + |  | - |  | + |  | - |  | + |  | - |
| Wild boar (Lower Austria 5) |  | + |  | - |  | + |  | - |  | + |  | - |
| Wild boar (Austria 1) |  | + |  | - |  | + |  | - |  | + |  | - |
| Wild boar (Austria 2) |  | + |  | + |  | + |  | - |  | + |  | - |
| Wild boar (Austria 3) |  | + |  | - |  | + |  | - |  | + |  | - |
| Wild boar (Austria 4) |  | + |  | - |  | + |  | - |  | + |  | - |
| Wild boar (Austria 5) |  | + |  | - |  | + |  | - |  | + |  | - |
| Wild boar (Romania) |  | + |  | - |  | + |  | - |  | + |  | - |
| Wild boar (Germany, Bad Kissingen) |  | + |  | - |  | + |  | + |  | + |  | - |
| Wild boar (Germany, Wolfsburg) |  | + |  | - |  | + |  | - |  | + |  | - |
| Wild boar (Germany, Rothenburg o.d. Tauber) |  | + |  | - |  | + |  | - |  | + |  | - |
| Wild boar (Germany, Leipzig) |  | - |  | + |  | + |  | - |  | + |  | - |
| Wild boar (Germany, Perleberg) |  | + |  | + |  | + |  | + |  | + |  | - |
| Wild boar (Germany, Landsberg) |  | + |  | - |  | + |  | - |  | + |  | - |
| Wild boar (Germany, Wiesbaden) |  | + |  | + |  | + |  | - |  | + |  | - |
| Wild boar (Germany, Everswalde) |  | + |  | - |  | + |  | - |  | + |  | - |
| Wild boar (Germany, Freisingen) |  | + |  | + |  | + |  | - |  | + |  | - |
| Wild boar (Germany) |  | + |  | - |  | + |  | - |  | + |  | - |
| Wild boar piebald (Germany) |  | + |  | - |  | + |  | - |  | + |  | - |
| Wild boar (Estonia 1) |  | + |  | - |  | + |  | - |  | + |  | - |
| Wild boar (Estonia 2) |  | + |  | - |  | + |  | - |  | + |  | - |
| Wild boar (Europe) |  | + |  | - |  | + |  | + |  | + |  | - |
| Wild boar (USA 1) |  | + |  | + |  | + |  | - |  | + |  | + |
| Wild boar (USA 2) |  | - |  | + |  | + |  | - |  | + |  | - |
| Wild boar (USA 3) |  | - |  | + |  | - |  | + |  | + |  | - |
| Class.… classification of the sample; W… wild boar; D… domestic pig; P… sum of wild boar and domestic pig | | | | | | | | | | | | |

**Supplementary Table 2:** Sequences and final concentrations of primers and probes, amplicon lengths, target genes and accession numbers of the real-time PCR assays.

| **Primer/Probe** | **Sequence (5' - 3')** | **Amplicon (bp)** | **Final concentration (nM)** | **Target gene** | **NCBI accession no.** | **Reference** |
| --- | --- | --- | --- | --- | --- | --- |
| **Singleplex assay for wild boar targeting the SNP on chromosome 9 (assay_Chr9_*_W_*)** | | | | | | |
| Chr 9 forward | GTAAGAAAATCTTAACCTAGCAAATGGGT | 73 | 12.5 | *Sus scrofa* | NC_010451.4 | [18] |
| Chr 9 wb reverse | AGGGAGTTTTTTGTTCTTACC**C**G |  | 200 | Intergenic |  |  |
| Chr 9 probe | ***6-FAM–*** CTCACAGGTGATGTGACT ***–MGB*** |  | 50 | Chromosome 9 |  |  |
| **Singleplex assay for domestic pig targeting the SNP on chromosome 9 (assay_Chr9_*_D_*)** | | | | | | |
| Chr 9 forward | GTAAGAAAATCTTAACCTAGCAAATGGGT | 74 | 62.5 | *Sus scrofa* | NC_010451.4 | [18] |
| Chr 9 dp reverse | CAGGGAGTTTTTTGTTCTTGGT**T**G |  | 800 | Intergenic |  |  |
| Chr 9 probe | ***6-FAM–*** CTCACAGGTGATGTGACT ***–MGB*** |  | 50 | Chromosome 9 |  |  |
| **Duplex assay for wild boar and domestic pig targeting the SNP on chromosome 1 (assay_Chr1_*_W_* and assay_Chr1_*_D_*)** | | | | | | |
| *NR6A1* forward | CCTGGGAACAGGGCTTCA | 67 | 1000 | *Sus scrofa* | AP009124 | [18] |
| *NR6A1* reverse | AAGCTCACCTGGAGGACAGTGT |  | 1000 | *NR6A1* gene |  |  |
| *NR6A1* wb probe | ***YY–*** CTCAC**C**GGGCTC ***–MGB*** |  | 200 | Chromosome 1 |  |  |
| *NR6A1* dp probe | ***6-FAM–*** CCAGCTCCTCAC**T**GG ***–MGB*** |  | 200 |  |  |  |
| **Singleplex assay for total pig targeting insertion/deletion on chromosome 7 (assay_Chr7_*_P_*)** | | | | | | |
| Forward | GGCAGGGAAGGTGTTTGTTATAGT | 119 (wb) | 300 | *Sus scrofa* | AB554586.1 | Based on [23] |
| Reverse | GACTGGCCTCTGTCCCTTGC | 411 (dp) | 300 | *Vertnin* (*VRTN*) gene |  |  |
| Probe | ***6-FAM–*** CGTGCCCAGAGCCTTGGCTCAT ***–BMN-Q530*** |  | 200 | Chromsome 7 |  |  |
| **Singleplex assay for domestic pig targeting insertion on chromosome 7 (assay_Chr9_*_D_*)** | | | | | | |
| Forward | GGCAGGGAAGGTGTTTGTTATAGT | 207 | 300 | *Sus scrofa* | AB554586.1 | Based on [23] |
| Reverse | CGGATCGTCAACCCACTGA |  | 300 | *Vertnin* (*VRTN*) gene |  |  |
| Probe | ***6-FAM–*** CGTGCCCAGAGCCTTGGCTCAT ***–BMN-Q530*** |  | 200 | Chromsome 7 |  |  |
| wb… wild boar; dp… domestic pig; MGB… minor groove binding quencher; YY… Yakima Yellow (fluorescent dye, absorbance maximum 530.5 nm, emission maximum 549 nm); FAM… (fluorescent dye, 6-Carboxyfluorescein, absorbance maximum 458 nm, emission maximum 515 nm); bold letters highlight subspecies-specific bases; underlined letters highlight mismatch bases; BMN-Q530… dark quencher | | | | | | |

## **Supplementary Material**

## **Verification of the qualitative results with a real-time PCR targeting a fragment of chromosome 7.** Analyses with the real-time PCR assay targeting a fragment of chromosome 7 were performed at Impetus GmbH & Co. Bioscience KG.

### Sample material was homogenized in a knife mill (Retsch GM200, Retsch Grindomix GM200; Retsch). Either twice 2 g or once 4 g of sample material were weighed out and 20 mL of CTAB puffer (2% (w/v) CTAB, 1.4 M NaCl, 0.1 M Tris, 20 mM EDTA, adjusted to pH 8.0 with 4 M HCl) and 20 µL of proteinase K (10 mg/mL, Roth) were added. After vortexing, the reaction mix was incubated at 60 °C under shaking (Hybridisation Oven, Gesellschaft für Labortechnik mbH (GFL), Burgwedel, Germany) for at least 3 h. Then the reaction mix was centrifuged (Swing-out rotor 5810R, Eppendorf, Hamburg, Germany) for 10 min at 3,220 x g at least, before 2 mL of the supernatant were mixed with 10 µL RNase A (5 mg/mL, Roth). The reaction mix was incubated for 15 min at 37 °C under shaking. After centrifugation (5430, Eppendorf) for 5 min at 20,817 x g at least, 900 µL of the supernatant were added to 600 µL chloroform in duplicate. After vortexing, the reaction mix was centrifuged (5430, Eppendorf) for 10 min at 20,817 x g at least. 625 µL of the aqueous phase were added to 500 µL isopropanol (abs.), mixed by inverting the tube and incubated at room temperature for at least 30 min. After centrifugation (5430, Eppendorf) for 10 min at 20,817 x g at least, the supernatant was discarded and the pellet was washed with 500 µL ethanol (70% (v/v) in water) twice. After careful removal of the residual ethanol the pellet was air dried at room temperature. Finally, the pellet was re-suspended in 50 - 100 µL 0.1 x TE-buffer (AppliChem; 1x Tris (10 mM Tris, 1 mM EDTA), pH 8.0) or water. To determine the DNA concentration and purity of the DNA isolates, the absorbance at 260 nm and 280 nm was determined using a spectrophotometer (Biotek Synergy HT, BioTek Instruments, Inc., Winooski, VT, USA). The DNA isolates were stored at - 20 °C.

### The real-time PCR assays targeting a fragment of the vertnin (VRTN) gene on chromosome 7 were developed at Impetus GmbH & Co. Bioscience KG. The primers and the probe were designed based on a previous study^23^. Primer and probe sequences, target genes, accession numbers (NCBI GenBank accession number), final concentrations of primers and probes used in the PCR assays and amplicon lengths are given in Supplementary Table 1. The primers and the probe were synthesized by Biomers (Ulm, Germany). Assay_Chr7P_ allows the selective detection of wild boar and domestic pig, assay_Chr7D_ is specific for domestic pig.

### Real-time PCR was carried out in an optical 96-well reaction plate (0.2 mL, Sarstedt, Nümbrecht, Germany; Thermo Fisher Scientific, Waltham, MA, USA or Bio-Rad Laboratories, Inc., Hercules, CA, USA) sealed with an optical adhesive film (Sarstedt, Thermo Fisher Scientific or Bio-Rad Laboratories Inc.) on an ABI 7500 Fast Real-Time PCR System (Applied Biosystems), a QuantStudio 5 Real-Time PCR System (Thermo Fisher Scientific), or on a CFX Connect Real-Time PCR Detection System (Bio-Rad Laboratories Inc.). Real-time PCR was performed in a total volume of 25 µL containing 12.5 µL Takyon Low Rox Probe MasterMix UNG (Eurogentec), forward and reverse primer (final concentration 300 nM each), probe (final concentration 200 nM), ultrapure water and 5 µL DNA isolate (20 ng/5µl). The temperature program consisted of an initial denaturation step at 95 °C for 5 min, followed by 45 cycles at 95 °C for 5 s, 60 °C for 30 s and 72 °C for 10 s.
